# The Value of a Modified Posterior Thigh Flap with Z-Plasty for Reconstruction of Ischial Tuberosity Pressure Ulcers: A Retrospective Single Center Analysis

**DOI:** 10.3390/life15020146

**Published:** 2025-01-22

**Authors:** Maximilian C. Stumpfe, Raymund E. Horch, Wibke Müller-Seubert

**Affiliations:** Department of Plastic and Hand Surgery and Laboratory for Tissue Engineering and Regenerative Medicine, University Hospital Erlangen, Friedrich-Alexander University Erlangen-Nürnberg (FAU), 91054 Erlangen, Germany

**Keywords:** ischial pressure sore, Posterior Thigh Flap, Z-plasty, fasciocutaneous flap, decubitus ulcer

## Abstract

Pressure ulcers are a persistent and growing challenge in modern medicine, with prevalence rates ranging from 3.4% to 32.4% globally. Demographic changes suggest an increasing number of patients at risk, emphasizing the need for effective prevention and advanced treatment strategies. These ulcers, particularly stages III and IV, often require surgical intervention due to severe tissue damage. Among the surgical options, the Posterior Thigh Flap has emerged as a reliable fasciocutaneous flap, frequently referred to as a “work-horse flap”, for defect coverage in the ischial region. To improve outcomes and minimize recurrences in weight-bearing areas, our clinic combined this flap technique with a Z-plasty modification to achieve redistribution of scar tissue to reduce localized pressure. In this retrospective study, six patients with seven ischial pressure ulcers were treated between 2003 and 2024 using this approach. Following debridement and wound conditioning with negative pressure therapy, defect coverage was performed. The results show clinically acceptable complication rates (42.9%) and a low recurrence rate (14.3%), with recurrences occurring no earlier than 12 months post-treatment. The Z-plasty effectively shifted scar zones away from high-pressure areas, reducing tension and recurrence risk. This study highlights the potential benefits of combining fasciocutaneous flaps with scar management techniques to improve long-term outcomes for ischial pressure ulcers and provides an innovative approach to treating ischial pressure ulcers.

## 1. Introduction

Pressure ulcers are ischemic damage to the skin and underlying tissue layers caused by prolonged external pressure, which particularly affects immobile, chronically ill patients. The annual incidence of pressure sores in America is between 1 and 3 million [1,2]. The proportion of pressure ulcers in hospitalized patients is stated in the literature to be between 5% and 15%. This rate can be markedly higher in intensive care units and certain long-term care facilities [3]. The prevalence rate worldwide is between 3.4% and 32.4% [4]. The development of decubitus ulcer is facilitated by prolonged pressure, friction, or shear forces, with skin and soft tissue defects occurring predominantly in the area of bony prominences. Normally, immobilized or bedridden persons are affected [5]. The sustained pressure leads to ischemia, which results in tissue damage [6]. An external pressure that is above the arterial capillary filling pressure (approx. 32 mmHg) and the venous capillary outflow pressure (approx. 8–12 mmHg) is already sufficient to cause the aforementioned tissue damage [7]. Preventive measures such as frequent changes in position, appropriate skin care and a balanced diet are therefore essential [8]. The National Pressure Ulcer Advisory Panel (NPUAP) categorizes pressure ulcers into five stages. Pressure ulcers progress from a reversible skin disorder in stage 1 to deeper tissue involvement in stage 2, with exposure of subcutaneous tissue. Stages 3 and 4 are characterized by deep ulcers involving muscles, tendons, bones, or body cavities, while stage 5 includes unclassifiable cases [9]. The treatment approaches vary depending on the severity and range from local wound care in early stages to surgical interventions in advanced stages [10].

Advanced treatment options include negative pressure wound therapy (NPWT) for wound conditioning [11]. The benefits of wound conditioning using NPWT and the positive influence on microbiological colonization are well documented in the literature [12,13].

Following debridement and negative pressure wound therapy, the defect must be reconstructed by a plastic surgeon. Thus, the established step-ladder approach based on plastic-reconstructive principles is also used in decubital surgery. However, split-thickness skin grafts are only indicated in exceptional cases, especially in the area of the lower extremities. Due to the limited pressure tolerance of split-thickness skin grafting, the value in the treatment of pelvic area pressure ulcers is low [14]. Therefore, skin grafting and sutures are reserved for inexperienced surgeons. Flaps, whereby a distinction is made between pivotal, rotational, and advancement flaps or, more rarely, free flap, are an adequate method for reconstructing tissue defects [11]. In practice, fasciocutaneous, myocutaneous, and perforator-based flaps are used as a standard [15].

**Figure 1:**
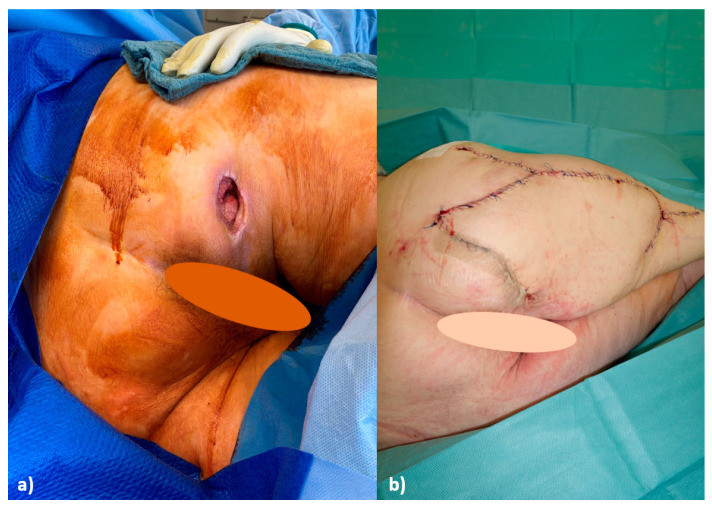
(**a**) Grade 4 decubitus ulcer on the right ischial tuberosity before initial debridement; (**b**) 1 week postoperatively after defect coverage using a Posterior Thigh Flap and Z-plasty.

**Figure 2:**
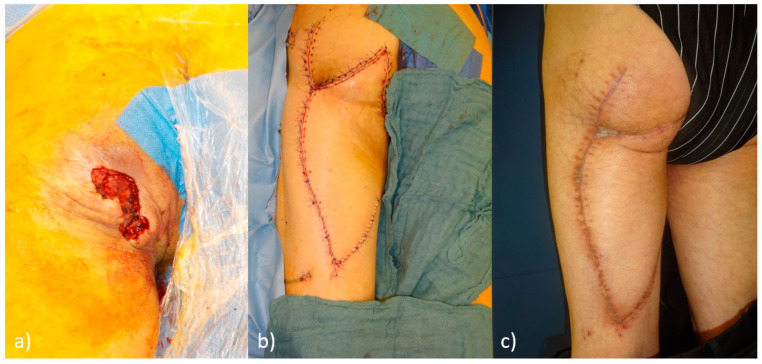
(**a**) Grade 4 pressure ulcer on the left side after initial debridement; (**b**) postoperative final result after defect coverage using a Posterior Thigh Flap and Z-plasty; (**c**) 4 weeks postoperatively after defect coverage.

In this context, a special mention should be made to the Posterior Thigh Flap (PTF) (Figure 1 and Figure 2). In decubitus surgery, the Posterior Thigh Flap, also known as the “work horse flap”, has proven to be particularly reliable and is used to cover defects in the ischial region. The PTF is perfused via the inferior gluteal artery and several perforators from the profunda femoris artery [16,17].

This study evaluates the efficacy and safety—including clinical outcomes, complication and recurrence rates—of a modified surgical approach combining the Posterior Thigh Flap with Z-plasty for reconstructing ischial tuberosity pressure ulcers.

## 2. Patients and Methodology

### 2.1. Patients

In the present study, a retrospective analysis of patients who were treated for pressure ulcers in the ischial region was performed. All patients who were diagnosed with a pressure ulcer and underwent surgical treatment using a Posterior Thigh Flap with combined Z-plasty and who were 18 years of age or older at the time of treatment were included in this study. We have reviewed the period from February 2003 to October 2024. The last patient was treated in October 2023. As part of the surgical treatment, all patients underwent radical debridement to remove all necrotic tissue. If necessary, a bone sample was taken to rule out osteomyelitis via histopathology. Following wound conditioning using NPWT and instillation of an antiseptic solution, Lavanid (0.4 mg/1 mL Lavasept, B. Braun Medical AG, Melsungen, Germany), the defect was covered using a Posterior Thigh Flap in combination with a Z-plasty.

Following defect coverage, intravenous antibiotics were administered based on the antibiograms of the current wound swabs.

The outpatient records were examined with a particular focus on complications, recurrences, and necessary follow-up procedures. Complications were recorded according to the Clavien–Dindo classification and also distinguished into minor and major complications. These include all deviations in healing processes, including minimal wound dehiscence, infections, and flap necrosis [18,19].

The time between defect coverage and completion of treatment was documented. A pressure ulcer in the area of a previously reconstructed and completely healed site in the area of the ischial bone was assessed as a recurrence. The absence of a further presentation at our clinic was assessed as recurrence-free.

The relevant data were evaluated using the Excel program (Microsoft, Redmond, WA, USA). Postoperative healing after surgical treatment was used as the primary outcome parameter.

This study was conducted retrospectively and anonymously, taking into account the institutional ethics committee and the Declaration of Helsinki and its subsequent amendments or comparable ethical standards. All patients have given their consent.

### 2.2. Surgical Technique

**Figure 3:**
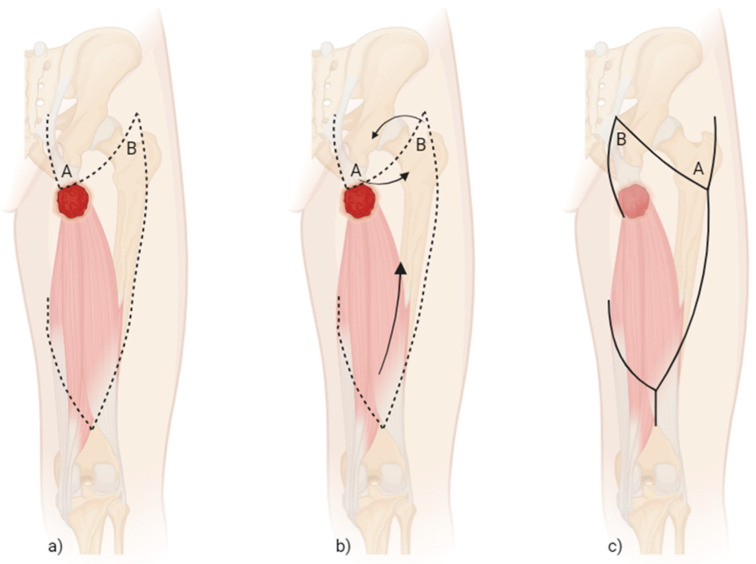
Schematic illustration of the preoperative marking (**a**), the rotation of the Z-plasty/two transposition flap (A and B) and the Posterior Thigh Flap (**b**), and the scar pattern after surgery (**c**); created by Maximilian C. Stumpfe.

As part of the preoperative marking, the anatomical landmarks of a Posterior Thigh Flap on the dorsal thigh with medial pedicle are used for orientation. It should be noted that the area of the tensor fasciae latae (TFL) flap and the trochanter must not be included in the operation. The caudal limit of the flap, which is to be lifted in the sense of a VY flap, is set at 10 cm proximal to the popliteal fossa. Furthermore, a back cut is made in the area of the medial border so that a skin bridge of 10 cm medially remains. Starting from the lateral border of the pressure ulcer, a Z-plasty is drawn, which includes the cranial lateral flap edge (Figure 3a,b). This procedure ultimately serves to distribute the scars in such a way that the maximum pressure load while sitting is not placed on the entire scar (Figure 3c).

Subfascial preparation of the flap is then performed according to the marking until tension-free closure. In addition, de-epithalization is performed in the area of the craniomedial flap to seal out the wound cavity and is fixed in depth in the area of the pressure ulcer. A total of three drains are inserted as part of the procedure. Suturing is performed using monofilament suture in a continuous suture technique and retentions sutures in a single-button technique. The sutures are removed on the 28th postoperative day. Additionally, consistent pressure relief of the flap over a period of four weeks is recommended. After the sutures have been removed, sitting training with a pressure-relieving cushions, for example, is recommended while the pressure on the flap is still being relieved. A gradual increase in sitting duration is recommended.

### 2.3. Statistical Analysis

Due to the small groups and the limited statistical power, only descriptive statistics were used. The analyses were performed with GraphPad Prism version 10 (GraphPad Software, Inc., San Diego, CA, USA).

## 3. Results

### 3.1. Demographic Data

In the present study, a total of seven pressure ulcers (85.7% stage IV; 14.3% stage III) in the area of the os ischium were treated in four male and two female patients between 2003 and 2024 using Posterior Thigh Flap with combined Z-plasty. One patient underwent a bilateral Posterior Thigh Flap with combined Z-plasty due to a bilateral stage IV pressure ulcer. The average age at the time of surgery was 57.1 ± 21.1 years (34.0–84.0 years). The mean duration of the pressure ulcer was 22.3 ± 14.6 months (7.0–36.0 months). Four pressure ulcers had previously been treated with debridement and NPWT. The remaining three pressure ulcers did not undergo prior surgical treatment and were treated conservatively instead. As part of the treatment at our department, all of the cases underwent extensive debridement, wound conditioning using NPWT (Solventum, 3M Kamen, Kamen, Germany), and instillation with Lavanid (0.4 mg/1 mL Lavasept, B. Braun Medical AG, Melsungen, Germany). In four cases (57%), the defect was closed on the right side, while in three cases (43%), it was closed on the left side.

In four cases, spina bifida was identified as the cause of the immobilization. In the remaining cases, paraplegia due to an accident was diagnosed. No serious secondary diseases were observed during the follow-up period. The main characteristics of the patients are summarized in Table 1.

### 3.2. Postoperative Course

The immediate postoperative course during the inpatient stay was free of complications in all patients. The three drains inserted as standard were all removed by the time of discharge, with an average of nine days after defect coverage. The average time to complete healing was four weeks. The minimum follow-up period for all patients ranged from 12 to 37 months. According to the Clavien–Dindo classification, postoperative morbidity during the outpatient consultations in our department corresponded to three grade 1 complications, represented a complication rate of 42.9%. All complications were minor complications. There were three small wound dehiscences that underwent conservative treatment and healed without complications.

In one case, there was a recurrence in the area of the ischial bone, which occurred twice. In one case, a grade 2 pressure ulcer manifested itself and was treated conservatively. The recurrence occurred 12 months after defect coverage. A further recurrence occurred 36 months after defect coverage. In all other treated cases, no recurrence was observed in the area of the ischial bone. This results in a recurrence rate of 14.3%.

## 4. Discussion

While conservative wound treatment with antiseptic procedures was initially the main focus for a long time, in 1938, Davis successfully reconstructed a pressure ulcer using a flap [20]. In 1956, Conway and Griffith presented the principles of modern wound reconstruction, which included bone and tissue debridement and subsequent defect closure [21]. The introduction of a myocutaneous flap from 1970 onwards significantly improved the success rate of flaps in decubitus surgery. This also resulted in an increase in the number of flap variants. At the same time, different flap techniques were established for different pressure ulcers [14].

Depending on the location of the pressure ulcer, various flap techniques are available to cover the defect. Gluteal flaps are the method of choice for presacral defects. For skin and soft tissue defects in the area of the greater trochanter, rectus femoris flaps and tensor fascia latae flaps are available. If the defect is in the area of the ischial tuberosity, it is possible to perform a biceps femoris flap or the Posterior Thigh Flap procedure described in this study [11].

In their publication, Homma et al. postulate the Posterior Thigh Flap as the first choice for grade III and IV pressure sores in the region of the ischial bone. They justify this by stating that this fasciocutaneous flap is closest to the ischial region, is versatile, and has a safe blood supply and low donor-site morbidity [22]. Djedovic et al. also recommend the Posterior Thigh Flap as the standard for defect coverage in ischial decubitus ulcers [23]. They rate the Posterior Thigh Flap as a reliable and simple flap that can be repositioned several times. In addition, they see advantages in the flap design outside pressure-prone areas and in the possibility of further reconstructions in the event of recurrences [23].

With regard to decubitus ulcers in the area of the ischial bone, the authors of this paper specify Posterior Thigh Flap as the “work-horse flap” and flap of first choice based on their clinical experience. The combination of Posterior Thigh Flap with a generous additional cranial Z-plasty, which was first mentioned by de Roche, has also proved to be successful in our approach [24]. The additional Z-plasty shifts the actual scar from the area of the ischial tuberosity medially to a more tension-free zone, thus minimizing the risk of recurrence.

The principle of Z-plasty is well known in plastic surgery for various applications, particularly since it was first described in the early 1800s and has been modified several times over time [25]. In the following, however, the following basic principle will be considered first: the Z-plasty is essentially a combination of two transposition flap procedures, which are applied at an angle of 60° to each other, thus lengthening the scar by approximately 75%. As a result, the scar can be displaced as described in our patient population [26].

A comparison of the present results with those in the literature shows that the complication rate for the patient group examined appears to be relatively high at 42.9%, although, in contrast to comparable literature data, all minor complications that could be successfully managed conservatively were included in this study. In this context, reference should be made to studies that report a rate from 7 to 42% [14,20,21,27,28]. A systematic review by Sameem et al. describes a complication rate of 18.6% for myocutaneous, 11.7% for fasciocutaneous, and 19.6% for perforator flaps [15]. It is not clear from the literature which criteria were used to define complications. In this study, all deviations from the normal postoperative course were defined as complications.

The recurrence rate, which is reported in the literature as ranging from 13 to 48.9 percent, appears relevant in this context [14,29,30]. The recurrence rate of 14.3% determined in the present study represents a comparatively low value for the Posterior Thigh Flap. The study by Sameem et al. also found no significant difference between the recurrence rates of fasciocutaneous, myocutaneous, and perforator flaps [15]. In their study, Homma et al. reported a recurrence rate of 30% for classic Posterior Thigh Flaps, which differs significantly from the recurrence rate determined in the present study [22]. Similarly, Yamamoto et al. reported a 27.8% recurrence rate for fasciocutaneous flaps in their paper [30]. In this context, it should be discussed that displacement of the scar is achieved by the Z-plasty, which reduces the recurrence rate in Posterior Thigh Flaps according to the authors. The period until the occurrence of a recurrence was also extended to a minimum of 12 months in our group. Hallock et al. showed a recurrence after 6 weeks with the conventional variant of the Posterior Thigh Flap [31].

The correct application of pressure relief measures is of crucial importance, both in preventive treatment and in postoperative therapy to prevent recurrences. Regardless of which flap surgery is ultimately performed, a recurrence is highly likely without further consistent prevention. Although the widespread opinion of a repositioning interval of two hours is no longer included in the latest guideline of the National Pressure Ulcer Advisory Panel (NPUAP), several studies show a benefit of shorter repositioning intervals compared to longer ones. Particular attention should be paid to the mattress. A reduction in the incidence rate from 7% to 10% was observed with a repositioning interval of 2 h compared to 4 h on a standard hospital mattress [9]. In a study in which the effects of intervals of four or six hours on a viscoelastic foam mattress were investigated, a reduction of 14% was demonstrated with shorter intervals [9]. In the case of alternating pressure mattresses, no difference in incidence was found [9]. Moore et al. emphasize the relevance of correct bed inclination and patient positioning [32]. In their study, they were able to demonstrate that a 30° sideways position in combination with a three-hour repositioning reduces the risk of developing pressure ulcers by 70% compared to a 90° sideways position with a six-hour repositioning interval. Furthermore, the authors postulate that a bed inclination angle that is as flat as possible can contribute to the reduction in shear and friction forces [32].

Like most comparable studies, this study is naturally limited due to its retrospective design and the small patient population. In addition, there is no uniform definition of the term recurrence in the literature, which leads to poor comparability between the surgical methods. In our opinion, prospective studies comparing the use of a classic Posterior Thigh Flap and its modification using Z-plasty should be sought in this regard.

## 5. Conclusions

The results presented in this study, with a comparatively low recurrence rate compared to the known results from the literature, demonstrate the possible benefit of an additional large-area Z-plasty in Posterior Thigh Flap surgery to shift the suture points away from the stress zone. In the authors’ opinion, this modification represents a practical and effective approach for reducing recurrence risks in ischial pressure ulcer reconstruction.

## Figures and Tables

**Table 1:** Characteristics of the patients.

Sex, Age	Localization	Cause	Co-Morbidities	Antibiotics	Stage	Complications	Recurrence
F, 84 yo	Right	Paraplegia	AHT, Adiposity	Ampicillin–Sulbactam	4	None	None
M, 52 yo	Left	Spina bifida		Piperacillin/Tazobactam	3	Minor	2 (12 and 36 months)
M, 73 yo	Right	Paraplegia	AHT	Piperacillin/Tazobactam	4	Minor	None
F, 39 * yo	Right	Spina bifida		Gentamycin, Clont	4	Minor	None
F, 39 * yo	Left	Spina bifida		Ciprofloxacin	4	None	None
M, 79 yo	Right	Paraplegia	AHT, Osteomyelitis	Piperacillin/Tazobactam	4	None	None
M, 34 yo	Left	Spina bifida	Osteomyelitis	Ampicillin–Sulbactam	4	None	None

yo = years old; AHT = Arterial hypertension, * = same patient; Stage = stage of pressure ulcer; F = female; M = male.

## Data Availability

The raw data supporting the conclusions of this article will be made available by the authors, without undue reservation.
